# Advancing health equity through cross-cutting approaches to health-related stigma

**DOI:** 10.1186/s12916-019-1282-0

**Published:** 2019-02-15

**Authors:** Gretchen L. Birbeck, Virginia Bond, Valerie Earnshaw, Musah Lumumba El-Nasoor

**Affiliations:** 1 Epilepsy Division, Department of Neurology, University of Rochester, Rochester, NY USA; 2 Chikankata Epilepsy Care Team, Chikankata Hospital, Mazabuka, Zambia; 3 Department of Global Health and Development, Faculty of Public Health and Policy, London School of Hygiene and Tropical Medicine, London, UK; 4 Zambart, School of Public Health, University of Zambia, Lusaka, Zambia; 5 Department of Human Development and Family Sciences, University of Delaware, Newark, DE USA; 6 Uganda Youth Coalition on Adolescent Sexual Reproductive Health Rights and HIV, Kampala, Uganda

## Abstract

Health-related stigma remains a major barrier to improving health and well-being for vulnerable populations around the world. This collection on stigma research and global health emerged largely as a result of a 2017 meeting on the “The Science of Stigma Reduction” sponsored by the US National Institutes of Health (NIH). An overwhelming consensus at the meeting was reached. It was determined that for stigma research to advance further, particularly to achieve effective and scalable stigma reduction interventions, the discipline of stigma research must evolve beyond disease-specific investigations and frameworks and move toward more unified theories of stigma that transcend individual conditions. This introduction reflects on the value of taking this cross-cutting approach from both a historical and current perspective, then briefly summarizes the span of articles. Collectively, the authors apply theory, frameworks, tools, interventions and evaluations to the breadth of stigma across conditions and vulnerabilities. They present a tactical argument for a more ethical, participatory, applied and transdisciplinary line of attack on health-related stigma, alongside promoting the dignity and voice of people living with stigmatized conditions. The collection homepage can be found at http://www.biomedcentral.com/collections/stigma.

## Introduction

The constitution of the World Health Organization includes the principle that “the enjoyment of the highest attainable standard of health is one of the fundamental rights of every human being without distinction of race, religion, political belief, economic or social condition [1].” Evidence shows that, globally, stigma is key in generating and perpetuating health inequities, despite the medical advances that make improved health possible [2] by deterring care-seeking and otherwise undermining individuals’ capacity to receive available care. Importantly, when care provision for stigmatized conditions is de-prioritized and/or overtly ignored, stigma also undermines investments in health. Consequently, there have been escalating calls to reduce stigma to promote health equity in a variety of disease contexts, including epilepsy [3], HIV [4], and mental illness [5]. This special collection of articles responds to these calls by articulating cross-cutting approaches to health-related stigma research and interventions in low and middle income countries (LMICs). It builds on an international effort to discuss the etiology and impact of stigma across conditions on the health of global citizens, while considering the methods and interventions that could be harnessed to measure and address stigma.

In recent decades, researchers have studied the risk factors and prevalence of health-related stigma, as well as how to measure stigma within certain disease contexts and populations, such as HIV, mental health and substance use. However, eliminating discrimination in healthcare settings requires time-bound targets and targeted funds, with resources allocated to programs and actions that are proven to work. The continually evolving burden of disease poses new challenges for the development of such interventions, with the added complexity that individuals may be affected by multiple stigmatized conditions, and/or potentially belong to stigmatized populations.

To date, most research to address health-related stigma has occurred within disease silos. Yet, theorists have highlighted significant similarities in the drivers, manifestations, and outcomes of stigma across health conditions [6]. Some researchers have also suggested that common approaches may be used to measure and intervene in stigma across health conditions [6, 7]. We are clearly at a tipping point in stigma research. New models for intervention research, novel approaches for studying intersectional stigma, and nimble research frameworks, which can be applied across different disease contexts, are needed if we are to make real headway in combatting some of the world’s most stubborn health problems. This special collection reflects the progress made in stigma research to date, as well as the evolving global health landscape and shifting disease burdens. Communities are now better poised than ever to actively partner in research, and increasingly, researchers recognize that interventions must be developed, evaluated, and implemented in collaboration with community members to be effective and sustainable [8].

Given the unfinished agenda of eliminating stigma to ensure health for all (particularly in LMICs), the rising burden of chronic, non-communicable diseases, and the fact that people with HIV are living longer and facing multiple stigmatizing conditions, there has been renewed interest in confronting health-related stigma in the global health context. This collection reflects the research challenges, priorities and opportunities addressed during the workshop, to catalyze new research approaches and collaborations and move this critical field forward.

### Progression of stigma theory: a historical perspective

**Fig. 1:**
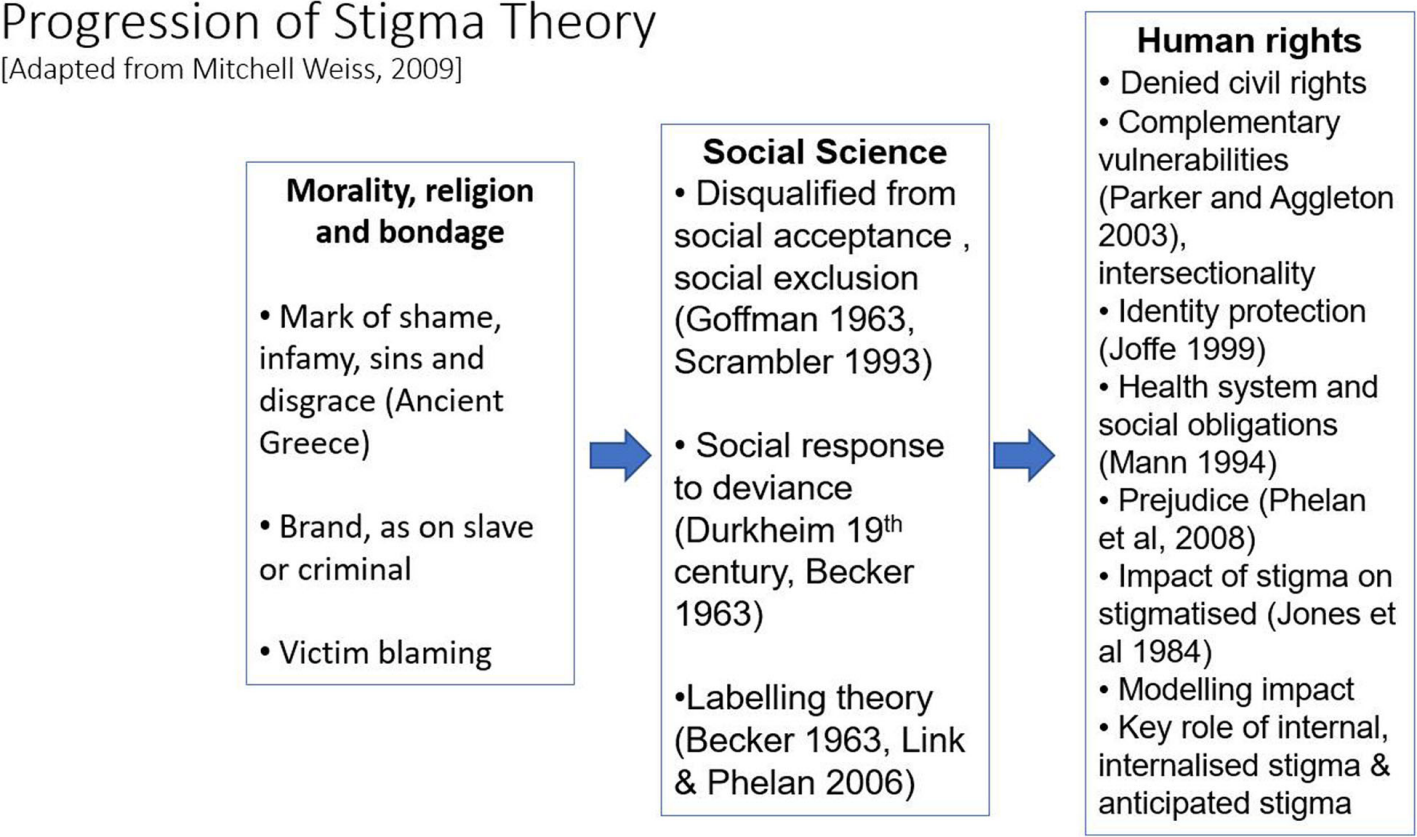
Progression of stigma theory, adapted from Weiss, 2008 [9]

A conclusion of the 2017 NIH meeting was that understanding stigma theory was critical for anti-stigma endeavors (for a brief summary of the progression of stigma theory, see Fig. 1). However, all too often, theory is either missing from endeavors or too heavily present to be useful beyond an academic network. Stigma theory emerged after World War 2, at a time when social science interest was “more in social theory than social policy or health policy [9],” and focused on the process of labeling and stigmatizing. This body of theory was developed in social science, pivoting around management of an identity affected by a “discrediting [10]” attribute and the concept of “deviance [11].” Both attributes and the “deviance” label were shown to endorse social norms and solidarity by labeling difference as a flaw, and as socially unacceptable. This leads to social exclusion and what Goffman termed “a spoiled identity [10].”

Unsurprisingly, the concept of “deviance” is now considered objectionable – not least because it overlooks the role of power [12]. Labeling, however, explicitly makes the link with power by exposing how social control and social construction drive fear about certain groups. Link and Phelan [13] break down the social process of stigma around labeling. First, ‘difference’ is identified and labeled, then a labeled person is linked to undesirable characteristics, followed by group labeling that separates “them” from “us”. The stigmatized group (“them”) subsequently experiences discrimination and loss of status. Power is exercised to reinforce this separation [13].

Inherent in labeling is the subsequent denial of civil rights, making stigma a human rights issue [9]. This recognition marked the shift from the academics of structural and social processes to considering psychological, health and behavioral outcomes and how stigma is experienced [14]. Tyler and Slater elaborate that, “redefining stigma has taken place NOT in sociology but in social psychology, medical and health research and, to lesser extent, law and criminology [15].” It became obvious that to really understand and resist stigma, consideration of the wider context and the political economy of stigmatization was needed [16]. As Deacon stated, “stigma is more than just an injury to an individual, but an indicator of the health of the social environment [17].” This includes what Deacon labels “the social landscape of prejudice [17].” Jonathan Mann, a key figure in advocating for HIV funding, called the sociopolitical response – including discrimination against HIV – the “third epidemic”, with the first epidemic being the spread of HIV and the second being AIDS as a disease. He recognized that meaningful and sustained social change against stigma will not occur unless social classification is challenged and social justice is pursued [15].

In the history of stigma theory, there are useful core concepts for health-related stigma. Jones et al. identified six dimensions of health conditions that could make them more or less stigmatized: concealability (hidden/visible), course of condition (and anticipated social consequences, disruptiveness (impact on social interactivity), aesthetic qualities (signs and symbols), origin (etiology and perceived blame), and peril (social danger). Key experiences of stigma were identified as devaluation, exclusion, and disadvantage [18]. The most common types of stigma are anticipated stigma (stigma that you fear may occur), enacted stigma (actions that occur against a person because of stigma) and internalized stigma (that which causes the individual to feel less of a person and “shame becomes a central possibility [10].” Each condition has both specific stigma features and the capacity to deepen stigma of another health condition.

Goffman closes his seminal book with an appeal for different disciplines to work together across stigmatised conditions. “Knowing what fields like race relations, aging and mental health share,” he urges us to examine the similarities and differences between a variety of disciplines [10]. It is only then, he claims, that we can come up with a “coherent analytic perspective”. This approach, called for more than 50 years ago, underlies this special collection.

### An overview of this collection on Stigma Research and Global Health

Each of the nine contributions in this collection on Stigma Research and Global Health offers stand-alone value, yet together they provide a comprehensive and complementary perspective on this important topic. A critical and shared perspective across the contributions is that to move toward meaningful and scalable interventions, stigma research must expand beyond the limitations inherent in addressing stigma associated with a single condition, to develop and embrace a unified or generic theory of its drivers and mechanisms, measurements for stigma and stigma interventions that transcends any individual stigmatized condition or identity.

In “Out of the silos: identifying cross-cutting features of health-related stigma to advance measurement and interventions,” van Brakel et al. [19] argue that their generic approach to stigma offers important opportunities for cross-cutting, synergistic research that will likely also be more cost-effective than investments in single-condition stigma reduction efforts. In “The Health, Stigma and Discrimination Framework,” Stangl et al. [20] take this idea even further by providing a global, cross-cutting framework to guide research, intervention development and policy on health-related stigma. Their framework is based upon theory, research and practice, and they offer illustrations of the framework applied across numerous conditions. Importantly, Stangl et al. note that identifying commonalities in the stigma process across conditions will amplify our collective ability to respond and scale-up. In “Challenges and opportunities in examining and addressing intersectional stigma and health”, Turan et al. [21] address the convergence of multiple stigmatized identities. Though it is clearly a common phenomenon when individuals with health-related stigma are viewed holistically rather than through a single-condition lens, to date, little research has been undertaken on this complex convergence. Turan et al. review the existing data, detail methodological gaps in the study of intersectional stigma, and suggest priority areas for future research to advance the field. In the context of intersectional stigma work, they also discuss the possibility that stigmatized individuals may gain resiliency and improved health and wellbeing through the solidarity of their own communities.

Three insightful systematic reviews offer further revelations about the present state of stigma research, and shine a light on priority areas for future study. In “A systematic review of multilevel stigma interventions: state of the science and future directions,” Rao et al. [22] identified 24 published examples of multilevel interventions, which mainly used an educational approach at the interpersonal and intrapersonal levels. They call for more rigorous study designs to address this gap. In “Implementation science and stigma reduction interventions in low and middle income countries: a systematic review” Kemp et al. [23] report on 35 published studies of evaluations of stigma reduction interventions in LMICs that offered at least one implementation outcome. They found that most of these studies examined acceptability and feasibility, none took a transdiagnostic approach to decrease stigma across multiple health conditions, and few included conceptual Implementation Sciences frameworks. They conclude that studies evaluating adoption, appropriateness, cost, fidelity, penetration and sustainability are needed, as well as more granular details of interventions. In “A scoping review of health-related stigma outcomes for high burden diseases in LMICs”, Kane et al. [24] explore HIV, mental illness, tuberculosis, epilepsy and substance abuse research. Their aim was to highlight commonalities across these conditions, including key moderators and mediators of stigma and health, and identify vulnerable and at-risk groups.

The systematic reviews in this collection are complemented by an Opinion piece on participatory research, and a Correspondence article on stigma in health facilities. Sprague et al. [25] articulate the imperative for participatory praxis, with an emphasis on the need for a shared starting point from the strengths and assets of the community. This would avoid further objectification, and rather enhance agency, dignitary and wellbeing, while also producing work that is more relevant, reliable and valid. This contribution recognizes the challenges in conducting participatory work, noting that, “*for community-engaged practice to become more than an ethical aspiration, structural changes in funding, training, publishing and tenure processes will be necessary.*” In the Correspondence article, “Stigma in health facilities: why it matters and how we can change it,” Nyblade et. al [26] provide a case study in which this cross-cutting approach has been applied to tackle stigma in healthcare facilities. They review the literature on stigma reduction strategies in health facilities, while also exploring the potential for facility-based strategies to occur across multiple conditions.

## Conclusion

As well as reviewing recent developments in research, theory, and intervention in health-related stigma in LMICs, this collection of articles provides a roadmap for researchers, healthcare providers, policymakers, community members, and other key stakeholders, to address the issue of stigma to enhance global health equity. Key guideposts on this roadmap include first deconstructing the silos that have, to date, stifled a fluid exchange of findings and innovations across disease contexts. Catalyzing cross-cutting approaches to research, theory, and intervention has the potential to spur more rapid and efficacious solutions to health-related stigma. Secondly, to understand and address this complex, multilevel social phenomenon, it is crictical to build transdisciplinary scientific teams. Solutions to health-related stigma will require expertise from public health, medicine, psychology, sociology, and anthropology, as well as other scientific disciplines. Thirdly, it is critical to partner with community members, providers, policymakers, and other stakeholders, to ensure that research and interventions are responsive, feasible, and efficacious. It is only through working together – across disease silos, disciplines, and scientist–community member boundaries – that we will be able to effectively address health-related stigma and enhance global health equity.

## Abbreviations

LMICs: Low and middle income countries
NIH: National Institutes of Health
